# Systematic Analysis of Transrectal Prostate Biopsies Using an Ink Method and Specific Histopathologic Protocol: A Prospective Study

**DOI:** 10.1155/2011/380249

**Published:** 2011-06-09

**Authors:** David Parada, Nahum Calvo, Karla Peña, Vanesa Morente, Rosana Queralt, Pilar Hernandez, Francesc Riu

**Affiliations:** ^1^Servei de Patologia i, Hospital Universitari Sant Joan de Reus, Tarragona 43206, Reus, Spain; ^2^Institut d'Investigació Sanitària Pere Virgili (IISPV), Universitat Rovira i Virgili, Tarragona, Reus, Spain; ^3^CRC Corporación Sanitària, Tarragona, Reus, Spain; ^4^Servei de Radiologia i, Hospital Universitari Sant Joan de Reus, Tarragona, 43206 Reus, Spain; ^5^Epidemiología, Estadística y Bioinformática, Institut d'Investigació Sanitària Pere Virgili (IISPV), Tarragona, Reus, Spain

## Abstract

*Background*. Transrectal prostate biopsy is the standard protocol for the screening for prostate cancer. It helps to locate prostatic adenocarcinoma and plan treatment. However, the increasing number of prostate biopsies leads to considerably greater costs for the pathology laboratories. In this study, we compare the traditional method with an ink method in combination with a systematic histopathologic protocol. *Methods*. Two hundred consecutive transrectal prostate biopsy specimens were received from the radiology department. They were separated into two groups: one hundred were processed as six different specimens in the usual manner. The other one hundred were submitted in six containers, the apex, base, and middle section of which were stained different colours. The samples subject to the ink method were embedded in paraffin and placed in two cassettes which were sectioned using a specific protocol. *Results*. The comparative study of the nonink and ink methods for histopathologic diagnosis showed no statistical differences as far as diagnostic categories were concerned (P  value < .005). The number of PIN diagnoses increased when the ink method was used, but no statistical differences were found. The ink method led to a cost reduction of 48.86%. *Conclusions*. Our ink method combined with a specific histopathologic protocol provided the same diagnostic quality, tumor location information as the traditional method, and lower pathology expenses.

## 1. Introduction

Biopsy specimens need to be appropriately histologically sampled if diagnoses are to be correct and complete. Inadequate sampling results in less informative diagnoses and misdiagnoses, while oversampling leads to wasted time and expenses. These issues are especially relevant to prostate needle biopsies because they comprise a significant portion of anatomic pathology practice and contain clinically relevant features that are often microscopically focal.

Systematic parasagittal sextant biopsy has been the standard protocol for many years in screening for prostate cancer [1–3], but this “standard method” has been modified to increase the probability of diagnosing prostatic adenocarcinoma and to allow for variation in the sampling and submission of prostate biopsies [4]. To improve the cancer detection rate, many prostate biopsy schemes have been extended to ten, twelve, or more cores per patient [5]. The increase in the number of prostate biopsies creates considerable extra expense for the pathology laboratory: consumables and work for technicians and pathologists' time. In this study, we analyzed the effectiveness of using an ink method for transrectal prostate biopsy combined with a histopathologic protocol that was similar to the routine histopathological method. 

## 2. Materials and Methods

### 2.1. Tissue Processing

For this study, radiologists submitted two hundred consecutive transrectal prostate biopsy specimens, from September 2009 to January 2010. Specimens were separated into two groups of one hundred samples each: the nonink group and the ink group. The nonink specimens were processed as six different specimens with one or two tissue cores. The apex and base were green and blue, respectively, while the middle section was not inked. The first one hundred were each embedded in paraffin blocks and sectioned until full-face tissue was observed, as usual. Three sections were obtained for histological examination. One slide of nonink material was given to the pathologist's staff. If they considered it to be necessary, they were free to obtain additional H-E (levels or a new H-E) and immunohistochemical studies.

The paraffin-embedded samples from the ink method were placed in two casettes (right and left) with at least six cylinders of material. The two blocks were sectioned using the following protocol. Level 1: 1 H-E + 3 unstained intervening slides for immunohistochemistry study +15 microns sections. Level 2: 1 H-E + 3 unstained intervening slides for immunohistochemistry study +15 microns sections. Level 3: 1 H-E + 3 unstained intervening slides for immunohistochemistry. Unstained sections were subject to immunohistochemical when it was required. The nonused unstained slides were discarded once a diagnosis had been made.

### 2.2. Histopathologic Review

The study focused on a series of specimens from 200 consecutive 18 gauge transrectal prostate biopsies that were performed at the Sant Joan University Hospital between 1 September, 2009 and 1 January, 2010. The diagnostic categories were, (1) benign prostate tissue, (2) prostatic intraepithelial neoplasia (high-grade PIN), (3) atypical glands suspicious for adenocarcinoma, and (4) prostatic adenocarcinoma. In each category, we evaluated the number of paraffin blocks, number of levels, and number of immunohistochemical studies.

### 2.3. Statistical Analysis

EPIDAT software was used to analyze the differences between the two protocols. A *P* value less than  .05 was considered significant. 

## 3. Results

### 3.1. Nonink Method

The histopathologic diagnoses in 100 consecutive cases of transrectal prostate biopsies were the following: benign prostatic tissue 56%, PIN 4%, atypical glands 3%, and adenocarcinoma 37%. A total of 600 initial histological examinations were carried out in each case, and 563 additional levels were required before a diagnosis could be made. Additionally, 66 immunohistochemical studies were performed to confirm the final diagnosis. The total number of H-E, level, and immunohistochemical studies was 1229. The total number of paraffin blocks was 600. The approximate cost of labor/reagents per block/slide/immunohistochemical studies was $22,734.76.

### 3.2. Ink Method

The histopathologic diagnoses in 100 consecutive cases of transrectal prostate biopsies were the following: benign prostate tissue 54%, PIN 8%, atypical glands 4%, and adenocarcinoma 34%. A total of 600 initial histological examinations were carried out, and no additional levels were required to make a diagnosis. Additionally, 54 immunohistochemical studies were performed to confirm the final diagnosis. The total number of H-E and immunohistochemical studies was 654. The total number of paraffin blocks was 200. The approximate cost of labor/reagents per block/slide/immunohistochemical study was $11,109.43.

### 3.3. Correlation between Methods

A comparative study between the nonink and ink method for histopathologic diagnosis showed no statistical differences in the diagnostic categories (*P*  value < .005) (Table 1). One interesting result was that the ink method resulted in twice as many diagnoses of PIN than the noninked method, but no statistical differences were found.  Table 2 shows the comparative use of resource, and Table 3 shows that, for each test and case studied by the nonink method, we required 0.53 levels, 0.82 immunohistochemical studies, and 0.33 paraffin blocks using the ink method. The cost reduction was 48.86%.

## 4. Discussion

Although systematic parasagittal sextant biopsy has been the standard protocol for many years, studies that use extended protocols have shown that it misses 10–30% of cancers [3, 6]. The ideal biopsy protocol still has to be determined [7, 8]. There are not any established guidelines for processing prostate biopsy specimens, and practices vary considerably. Our study aims to determine which histological procedure can be used with confidence in the general pathology laboratory. We have chosen to address this question using two different methods: the first is a traditional procedure used in routine pathology laboratories, while the second—the ink method—consists of cylinders stained with colored ink depending on prostate location and a specific histopathologic protocol. Our results showed that the ink method had the same diagnostic quality and gave the same information about prostatic adenocarcinoma.

Using our protocol, the biopsies from the right and left side of the prostate are placed in two paraffin blocks and three HE staining sections of each block are obtained for diagnosis. In this way, pathology expenses are cut by more than half and the information provided about location is the same. The protocol also permits three immunohistochemical markers when necessary, which presents no problems of the loss of paraffin-embedded material.

Previous research has been carried out on the use of the ink method for prostate biopsy [9, 10], and results show that costs can be reduced with the same information about tumor location provided. However, this research focused only on the specific location of the prostate specimen. In our study, we used a specific histopathologic protocol coupled with the ink method, and, in terms of diagnostic category, the results were not different from those of the traditional method. The detection of prostatic intraepithelial neoplasia increased, probably because none of the material studied for diagnosis was lost. On the other hand, when multiple prostate biopsy cores are embedded in a single block, probably less tissue is evaluated because it is difficult to embed all cores in a single plane for optimal tissue presentation [11]. However, with proper handling, multiple cores per cassette seems to be reasonable. Finally, by reducing the number of blocks/slides from six to two, the potential savings could be in hundreds of million per year. Saving has also been shown when the containers are reduced from 12 to 6 [12].

In conclusion, the application of tissue-marking ink in prostate biopsies can prevent valuable information about tumor location from being lost. The combination of the ink method and specific histopathologic information reduces the time and cost of processing biopsies and can still guarantee reliable results.

## Figures and Tables

**Table 1:** Diagnostic proportion differences between nonink and ing groups.

	% nonink	% ink	Difference %
			**P** value
Adenocarcinoma	37.0	34.0	.77
PIN	4.0	8.0	.37
Atypical glands	3.0	4.0	1.00
Benign	56.0	54.0	.89

*P*  value < .005 for differences among all categories.

**Table 2:** Comparative use of resources between nonink and ink groups.

	Number	% nonink	% ink	Difference %
				*P* value
H-E levels	863	65.2	34.8	.0001
Immunohistochemistry	120	55.0	45.0	.16
Block	800	75.0	25.0	.0001
Levels + immunohist.	983	64.0	36.0	.0001

*P*  value < .005 for difference among all categories.

**Table 3:** Relation of the use of resources for each method and sample (mean values).

	Nonink	Ink	Ratio nonink/ink	Ratio ink/nonink
H-E levels	5.63	3.00	1.88	0.53
Immunohistochemistry	0.66	0.54	1.22	0.82
Block	6.00	2.00	3.00	0.33
Levels + immunohist.	6.29	3.54	1.78	0.56
